# Low empathy-like behaviour in male mice associates with impaired sociability, emotional memory, physiological stress reactivity and variations in neurobiological regulations

**DOI:** 10.1371/journal.pone.0188907

**Published:** 2017-12-04

**Authors:** Giovanni Laviola, Francesca Zoratto, Danilo Ingiosi, Valentina Carito, Damien Huzard, Marco Fiore, Simone Macrì

**Affiliations:** 1 Reference Centre for Behavioural Sciences and Mental Health, Istituto Superiore di Sanità (ISS), Rome, Italy; 2 Institute of Cell Biology and Neurobiology, National Research Council of Italy (CNR), Rome, Italy; 3 Laboratory of Behavioural Genetics, Brain Mind Institute, Ecole Polytechnique Federale de Lausanne (EPFL), Lausanne, Switzerland; Technion Israel Institute of Technology, ISRAEL

## Abstract

Deficits in empathy have been proposed to constitute a hallmark of several psychiatric disturbances like conduct disorder, antisocial and narcissistic personality disorders. Limited sensitivity to punishment, shallow or deficient affect and reduced physiological reactivity to environmental stressors have been often reported to co-occur with limited empathy and contribute to the onset of antisocial phenotypes. Empathy in its simplest form (i.e. emotional contagion) is addressed in preclinical models through the evaluation of the social transmission of emotional states: mice exposed to a painful stimulus display a higher response if in the presence of a familiar individual experiencing a higher degree of discomfort, than in isolation. In the present study, we investigated whether a reduction of emotional contagion can be considered a predictor of reduced sociality, sensitivity to punishment and physiological stress reactivity. To this aim, we first evaluated emotional contagion in a group of Balb/cJ mice and then discretised their values in four quartiles. The upper (i.e. Emotional Contagion Prone, ECP) and the lower (i.e. Emotional Contagion Resistant, ECR) quartiles constituted the experimental groups. Our results indicate that mice in the lower quartile are characterized by reduced sociability, impaired memory of negative events and dampened hypothalamic-pituitary-adrenocortical reactivity to external stressors. Furthermore, in the absence of changes in oxytocin receptor density, we show that these mice exhibit elevated concentrations of oxytocin and vasopressin and reduced density of BDNF receptors in behaviourally-relevant brain areas. Thus, not only do present results translate to the preclinical investigation of psychiatric disturbances, but also they can contribute to the study of emotional contagion in terms of its adaptive significance.

## Introduction

Empathy enables individuals to share the affective feeling of others, to anticipate their actions [1, 2] and it stimulates prosocial behaviour [3–5]. Lipps, who provided the original definition of the term [6], described empathy as a process by which “the perception of an emotional gesture in another directly activates the same emotion in the perceiver, without any intervening labelling, associative or cognitive perspective-taking processes” [7, 8]. Empathy is thought to have evolutionary precursors that allow animals to share emotional states even without being able to identify the source or to comprehend the causality of the emotion aroused in the other. This particular phenomenon was originally described in social animals by ethologists, who called it “Stimmungsübertragung” [9], translatable as “emotional contagion” or “mood induction” [1]. This simpler form of empathy, which involves the adoption of another’s emotional state, is thus regarded as a phylogenetic precursor or a physiological prerequisite for more complex forms of empathy (i.e. those resulting from the interaction between emotional and cognitive processes [1, 10]) and it is considered at the core of all empathic behaviours [11, 12].

The construct of empathy is amenable to being addressed within the evolutionary adaptive framework offered by the Tinbergen four whys [13]: with respect to evolutionary adaptive considerations, the ability to empathize affects an individual’s behaviour toward others and the quality of social relationships, ultimately influencing individual fitness [14]; from an ontogenetic perspective, empathy is an important part of social development and its maturation co-occurs with emotional and cognitive processes; with respect to phylogeny, different levels of empathy have been identified in several taxa, ranging from rodents to elephants and chimpanzees [15]; finally, the proximate causations and some of the fundamental biological mechanisms governing empathy across species have been identified [8].

Empathy for pain, defined as the ability to share the emotions of others who are exposed to painful stimuli, is considered a key evolutionarily conserved form of empathy [2, 8, 16, 17] and it has been explored by means of several models based on emotional contagion in both humans and non-human animals [7, 18–20]. For example, Langford and collaborators [19] demonstrated that pain experience could be socially transmitted in rodents. Specifically, the authors demonstrated that the presence of a conspecific experiencing pain influenced pain perception in a related (cagemate or sibling) laboratory mouse. Smith and collaborators [21] recently extended these original data by demonstrating that laboratory mice can socially transmit different forms of pain, induced through diverse modalities. Furthermore, the authors demonstrated that social transmission of pain does not necessarily involve visual information but can occur in the presence of olfactory cues [21].

Just as these studies demonstrated the presence of emotional contagion in non-primate animals, so also several authors started detailing the neurobiological mechanisms modulating empathy. For example, Sivaselvachandran and collaborators [15] summarized preclinical evidence reporting the involvement of specific brain areas in the modulation of empathy-like behaviour: prefrontal cortex, anterior cingulate cortex, ventral tegmental area, thalamus and amygdala [15]. This preclinical evidence is also paralleled by clinical investigations that allowed the identification of a neural circuitry controlling a plethora of behaviours including empathy, sociability and emotional memory [22]. Adopting a multivariate approach, Newman (1999) described the “social behaviour network”, a structure consisting of brain areas (lateral septum, prefrontal cortex, extended amygdala, midbrain, preoptic area, several hypothalamic nuclei and other limbic structures), neuroendocrine mediators (oxytocin, vasopressin, opioids, cannabinoids and neurotrophins) and peripheral systems (hypothalamic-pituitary-adrenal, HPA, axis), that controls the exhibition of socially-relevant phenotypes [22].

While empathy influences individual development at many levels, defects in this fundamental process have been involved in a series of neuropsychiatric disturbances [23]. Specifically, a general lack of empathy is explicitly indicated as a diagnostic criterion or feature of five disorders belonging to four distinct categories, as described in Section II of DSM-5: Conduct Disorder (category: Disruptive, Impulse-Control, and Conduct Disorders); Antisocial Personality Disorder and Narcissistic Personality Disorder (category: Personality Disorders); Intellectual Disability (category: Neurodevelopmental Disorders); Major/Mild Frontotemporal Neurocognitive Disorder (category: Neurocognitive Disorders).

Among the abovementioned conditions, here we addressed in particular conduct disorder (CD). Notably, a general lack of empathy (callousness [24]) has been proposed to constitute a core symptom in a subgroup of individuals characterized by CD [25]. Callous-unemotional traits (CU traits) are often found in individuals who engage in antisocial behaviour to reach a goal and callousness is also one of the hallmarks of psychopathy [26]. CD, a highly prevalent (2–10%) disturbance in the child and adolescent population, is characterized by excess aggression, limited expression of prosocial behaviours, reduced emotionality, shallow or deficient affect, diminished physiological stress reactivity, patterns of social norm violation and antisocial behaviours [25, 27, 28]. Furthermore, the revised DSM-5 diagnostic criteria for CD includes a specifier for “limited prosocial emotions” that targets CU traits found in a subset of CD individuals [26, 29, 30]. Conduct problems, especially in case of an early onset, are thought to be predictive of an increased risk of educational disruption, substance-related disorders, criminal behaviour and antisocial personality disorder in adulthood [31]. Our primary focus on CD did not solely rest upon lack of empathy but on the constellation of symptoms we addressed in our experimental model (see below for further details). Thus, we originally hypothesised that reduced empathy may represent a proxy for CD, and tested this hypothesis through the evaluation of different phenotypes.

**Fig 1:**
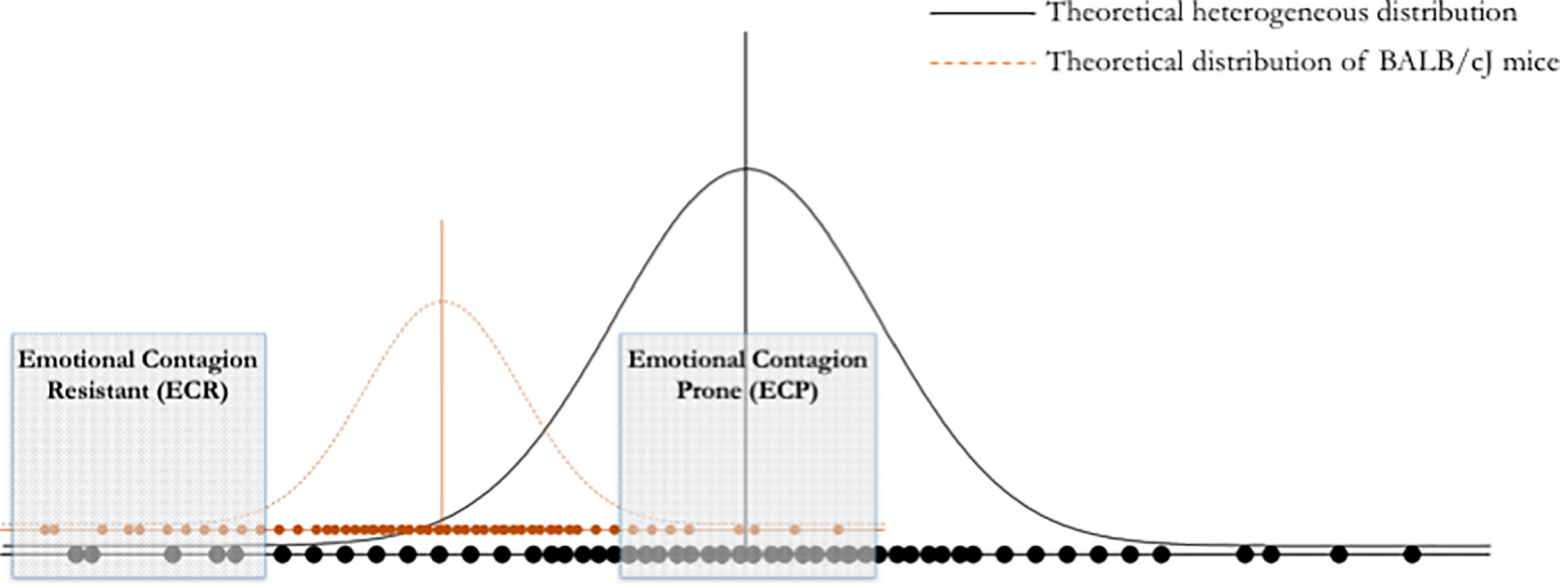
Theoretical rationale behind the selection of BALB/cJ mice as study subjects. Based on literature data indicating that BALB/cJ mice exhibit very low levels of social behaviour and on the hypothetical association between social behaviour and emotional contagion, we selected the upper and lower quartiles of BALB/cJ mice as our study population. The full line indicates the theoretical distribution of emotional contagion in a heterogeneous population of different mouse strains; the dashed line represents the theoretical distribution of BALB/cJ mice and the shaded rectangles represent our study population characterised by emotional contagion resistant (ECR; left rectangle) and prone (ECP; right rectangle) populations. Importantly whilst emotional contagion resistant theoretically represent an extreme population, emotional contagion prone subjects shall locate around the average of the general population.

Ultimately, the construct of empathy constitutes a central node in biology whereby it combines evolutionary-adaptive considerations with neurobiological and biomedical implications. In the present study, we aimed at demonstrating the association between a rudimentary form of empathy (emotional contagion) and the other components of the social behaviour network. Furthermore, we aimed at investigating selected neurochemical correlates of this association. To this aim, we first selected two subgroups of mice exhibiting very low or average levels of emotional contagion addressed through a paradigm based on the evaluation of the social transmission of emotional states and then performed a series of experimental tests mapping onto social behaviour, aggression, pain perception and response, memory and punishment learning. We hypothesized that low levels of emotional contagion correlated with reduced sociality, increased aggression and impaired performance in punishment learning. We then investigated the extent to which the observed alterations co-occurred with variations in hormonal stress reactivity (plasma corticosterone in baseline conditions and in response to restraint stress) and neurobiological regulations (peptides and receptors of brain derived neurotrophic factor, BDNF, oxytocin and vasopressin) in the anatomical networks involved in empathy-like behaviour. To leverage a population exhibiting very low levels of emotional contagion, we rested our study upon mice belonging to the BALB/cJ mice. This strain has been shown, in independent studies, to exhibit extremely low levels of social behaviour (e.g. [32]). Under the assumption that social behaviour correlates with emotional contagion, we inferred that BALB/cJ mice would locate on the left-end side of the Gaussian curve describing the frequency distribution of emotional contagion. Specifically, assuming a heterogeneous population of mice constituted by several different strains, the bell-shaped curve of BALB/cJ mice shall cluster on the left-end of the Gaussian distribution. Within BALB/cJ, those exhibiting very low levels of emotional contagion should reside in the extreme left-end of the distribution describing the heterogeneous population; conversely BALB/cJ mice characterised by very high levels of emotional contagion may be closer to the centre of the theoretical distribution. Ultimately, the selection of BALB/cJ mice was meant to allow the comparison between subjects exhibiting very low absolute values of emotional contagion and subjects clustering around the mean of a general population. These theoretical considerations are sketched in **Fig 1**.

We then tested whether very limited levels of emotional contagion in mice are associated, as reported in humans, with increased aggression, limited sociability, reactivity to punishment, and physiological stress reactivity; furthermore, we analysed some of the neurochemical mechanisms associated with empathy in humans (EXPERIMENT 1). To test whether inter-individual differences in emotional contagion are dependent on baseline differential pain sensitivity, an independent batch of experimental mice have been tested for pain reactivity to a chemical agent (EXPERIMENT 2). Finally, to further our knowledge concerning the mechanisms favouring emotional contagion, we assessed the relevance of visual and olfactory cues (EXPERIMENT 3).

## Materials and methods

### Ethics statement

All experimental procedures were approved by Institutional Animal Survey Board on behalf of the Italian Ministry of Health (licence DM 05/2014 B to G. Laviola) and performed in full accordance with the Directive 2010/63/EU on the protection of animals used for scientific purposes and Italian law (Legislative Decree 26/2014). All efforts were made to minimize animal suffering and to reduce the number of animals used.

### Animals and rearing conditions

Experimental subjects were male BALB/cJ mice (USA, JAX^TM^ Mice stock n° 000651) provided through Charles River. Upon arrival, at 25 days of age, BALB/cJ mice were housed in 33 × 13 × 14 cm Plexiglas boxes in groups of four. We also purchased C57BL/6J mice from Charles River (Italy) to be used in the resident-intruder test (see below). C57BL/6J mice were housed in groups of 6 in 42 × 17 × 14 cm Plexiglas boxes, in the same housing room as BALB/cJ mice. Animals had access to water, environmental enrichment in the form of shelter material and food *ad libitum* (Mucedola, Settimo Milanese, Italy). The animal room was maintained under an inverted 12:12 h L/D cycle (lights on at 07:00 pm). Temperature (22 ± 1°C) and humidity (42–52%) were monitored and kept constant.

### Experiment 1: Outline

**Fig 2:**
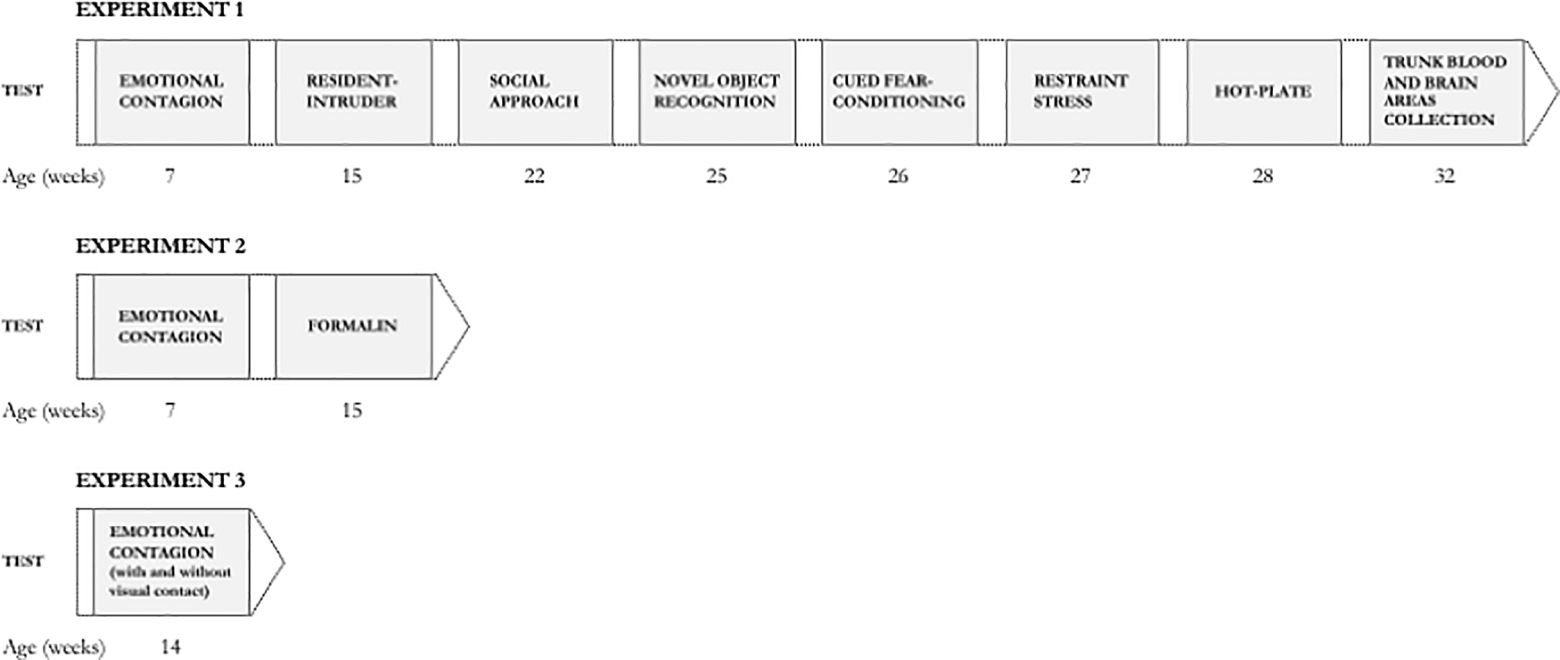
Timeline of the study. Timing, expressed in weeks, of the behavioural and physiological investigations in male BALB/cJ mice.

Forty BALB/cJ male mice were used for this experiment. Upon arrival, mice were left undisturbed for 18 days; then, they were all subjected to a paradigm for the evaluation of the social transmission of emotional states (i.e. emotional contagion test) at seven weeks of age. This first test allowed us to identify and select, within the 30 mice that acted as Observers (for details, see the paragraph “Emotional contagion test”), two subgroups of mice exhibiting extreme values (very high or very low) of emotional contagion. Specifically, the upper quartile was defined as the “Emotional Contagion Prone” (ECP) group while the lower quartile was defined as the “Emotional Contagion Resistant” (ECR group). To examine whether low levels of emotional contagion generalize to other behavioural measures, we performed an extensive phenotypic characterization of ECP and ECR mice (summarized in **Fig 2**): resident-intruder test at 15 weeks; social approach test at 22 weeks; novel object recognition test, cued fear-conditioning test, response to restraint stress, hot-plate test respectively at 25, 26, 27, 28 weeks. Four weeks after the end of behavioural testing (week 32), trunk blood and brain areas were collected.

### Experiment 1: Behavioural tests

#### Emotional contagion test

To investigate inter-individual differences in emotional contagion, we evaluated the extent to which a painful experience can be socially transmitted between individuals [19]. Pain has been induced through the subcutaneous injection of a small volume of formalin (25 μl), diluted in vehicle (0.9% saline solution), into the plantar surface of the animal’s hind paw (see [33, 34] for details). {Gioiosa, 2009 #195;Rosland, 1990 #220}.

**Fig 3:**
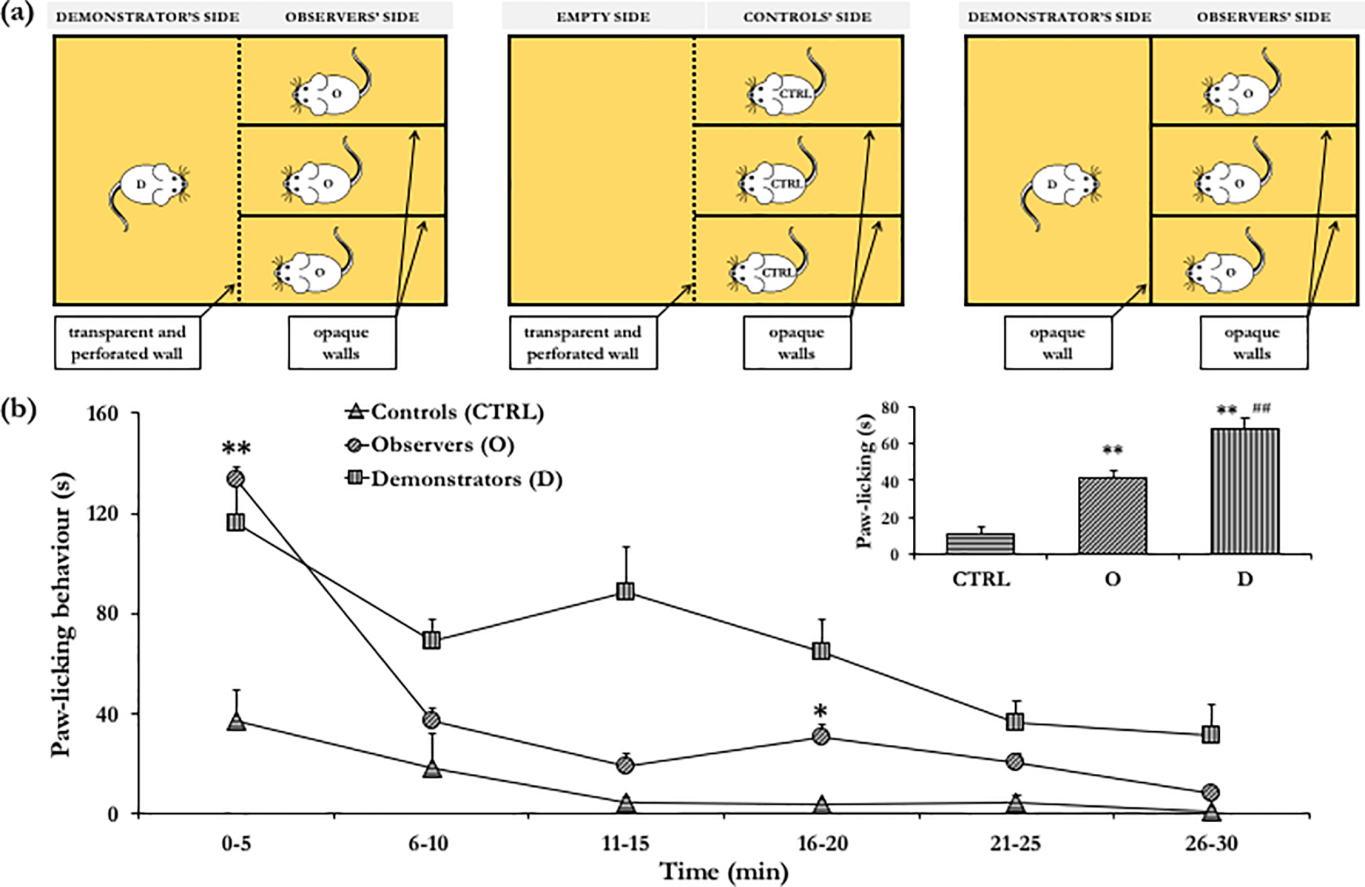
The emotional contagion test. **(a) Schematic representation of the experimental apparatus. (left)** The Demonstrator mouse (D) is injected with 1% formalin concentration whilst the 3 Observers (O) mice with 0.2% formalin concentration. O mice are then tested in visual and olfactory contact with a D mouse. This setting was used in EXPERIMENTS 1, 2, 3. **(middle)** Control individuals (CTRL) are injected with 0.2% formalin concentration and tested in the absence of a D mouse. This setting was used in EXPERIMENT 1. **(right)** The D mouse is injected with 1% formalin concentration whilst the 3 O mice with 0.2% formalin concentration. O mice are then tested in the presence of a D mouse providing olfactory but not visual cues. This setting was used in EXPERIMENT 3. **(b) Paw-licking behaviour in mice injected with different formalin concentrations in EXPERIMENT 1.** Controls (CTRL) curve: male mice tested individually for paw-licking response to injection in a rear paw with 0.2% formalin concentration. Observers (O) curve: mice tested socially for paw-licking response to injection with 0.2% formalin concentration (i.e. in the presence of a Demonstrator mouse injected with 1% formalin concentration). Demonstrators (D) curve: mice tested socially for paw-licking response upon 1% formalin concentration. ** p < 0.01, O vs CTRL during interval 0–5; * p < 0.05, O vs CTRL during interval 16–20. **(inset)** Integral response of CTRL, O and D mice, obtained as the average of the values constituting **Fig 3B**. ** p < 0.01, significantly different from CTRL; ^##^ p < 0.01 significantly different from O.

For the emotional contagion test we used a custom-made apparatus consisting of an opaque plastic box (20 × 20 × 30 cm) subdivided in two equally-sized sectors by a transparent and perforated Plexiglas partition which allowed olfactory, acoustic and visual communication (see **Fig 3A** for a schematic representation of experimental apparatus). One-half of the apparatus was the Demonstrator’s side. The other half, the Observers’ side, was further subdivided in three compartments by opaque plastic walls, which prevented visual communication. Thus, Observers (O) were in visual contact only with the Demonstrator (D) but not with each other. Before the test, mice were habituated to the apparatus for 10 minutes during three consecutive days.

The test consisted of two phases. Four subjects (cagemates, 1 D, 3 O) were placed in the respective compartment for 10 minutes prior to formalin injection (baseline). Subsequently, D and O mice were briefly removed and injected with formalin (the D mouse with 1% formalin concentration; the three O mice with 0.2% formalin concentration) and returned to the assigned compartment for 30 minutes (test phase). The test was performed minimizing gradients in light, temperature, sound and other environmental conditions. Between sessions, the apparatus was cleaned with 30% ethanol/water solution. To demonstrate that the presence of the Demonstrator influenced pain perception in Observers, a third group of control (CTRL) individuals was injected with 0.2% formalin concentration in the absence of the Demonstrator. Each mouse was assigned to only one group (O, D or CTRL) and used in this test just one time. The duration of paw-licking behaviour was scored in Observers, Demonstrators and Control subjects.

#### Resident-intruder test

To investigate levels of aggressive, affiliative and non-social behaviours, we tested BALB/cJ mice in their home cage for response towards an unfamiliar C57BL/6J male mouse [35], which weighed slightly less than resident BALB/cJ mice. After 1 week of individual housing (33 × 13 × 14 cm Plexiglas box) resident BALB/cJ mice were allowed to interact for 10 min, for 3 consecutive days, with the C57BL/6J male mouse. Testing was conducted in an experimental room adjacent to the housing room. For the duration of the test, the standard wire lid was replaced with a transparent cover. In accordance with the procedure described by Velez and colleagues (2010), each resident mouse was confronted with the same C57BL/6J male mouse throughout the 3 days of testing. After each encounter, the C57BL/6J mice were returned to their home cage [36].

Behavioural patterns exhibited by the resident mouse BALB/cJ towards the intruder mouse C57BL/6J were subdivided in the two categories of affiliative and aggressive behaviours and scored in frequency and duration [37]:

- affiliative behaviours: anogenital sniffing (sniffing the anogenital area of the intruder mouse), investigating (sniffing movements at the head and body of the intruder mouse except its anogenital region), allogrooming (grooming the intruder mouse), received grooming (being groomed by the intruder mouse);
- aggressive behaviours: latency to the first attack (the time from the beginning of the test session to the first biting attack), attack (biting attack of resident mice), fixating (only the forepaws are moved towards the intruder mouse resulting in a fully stretched posture towards the latter), aggressive grooming (violent grooming on the back of the intruder mouse), chase (the animal goes after the intruder mouse), lateral threatening (slowly moving in a sideways direction to or around the intruder mouse), upright posture (the mouse stands on its rear paws in reaction to approach or upright of the intruder mouse), clinch (very rapid rolling, jumping and biting of both animals that are in close contact), keeping down (standing over the intruder mouse), keeping off (kicking movements with one of the rear paws towards the intruder mouse), mounting (the resident mounts the intruder mouse as in a male female encounter).

#### Social approach test

Social behaviours in an unfamiliar environment were investigated in test mice using a three-chambered apparatus [38]. In this test, focal experimental subjects are allowed to explore a three-partitioned apparatus consisting of a central zone and two target compartments containing social or inanimate stimuli. Briefly, the experimental subject is allowed to first display a preference for a conspecific over an inanimate object (sociability) and then for an unfamiliar subject over a familiar one (social preference). Target mice were male BALB/cJ, of the same sex and age as the experimental subjects. The social approach test procedure consisted of three phases: habituation, sociability and preference for social novelty. In the first phase, mice were individually allowed to freely explore for 20 minutes the entire apparatus. In the subsequent phase (sociability), experimental subjects were briefly confined to the central compartment and a novel target mouse (unfamiliar mouse named stranger 1) was placed inside a wire enclosure in one of the two chambers. Stranger 1 was previously habituated to the wire enclosure for 5 min/day for 3 consecutive days. An identical empty wire enclosure was placed in the opposite side. After both stimuli were positioned, the test mouse was free to move throughout all three chambers for 10 min. Preference for social novelty phase (10 min) began immediately after the sociability phase. Before sociability and social novelty phase, focal mice were confined to the central compartment while a new novel target mouse (stranger 2) was placed in the wire cage that was empty during the sociability phase. The stranger 1 remained in its wire cage. Stranger 1 and stranger 2 originated from different cages and had never been in physical contact with the test mice or to each other [39]. Spatiotemporal variables (i.e. time spent in each compartment and number of crossing between compartments) were scored in test mice; behaviours of target mice were not scored.

#### Novel object recognition test

We used the novel object recognition (NOR) test to investigate the recognition memory of test mice [40]. The apparatus consisted of a custom-made black Plexiglas open field (42 × 42 × 30 cm). Stimuli were two different objects. The open field was located in a dedicated room. NOR test consisted of three phases: habituation, acquisition and retention. Before the test day, mice were individually habituated for 10 min/day for 4 consecutive days in the empty open field. During the acquisition phase, after two different objects were placed in the middle of the arena, without the possibility to be moved, each test mouse was introduced into the open field where it was free to move and explore for 10 min. The retention phase (5 min) was performed 1 h after the acquisition phase. Each mouse was placed back into the same arena with one same object (A or B), as during the acquisition phase and a new object (named C), both in the same location of acquisition phase. The objects were plastic bowls, different in colour and form. Objects presented during acquisition phase and the new one presented during retention phase were randomized between animals. At the end of each phase, the open field and the objects were cleaned with 30% ethanol/water solution. The duration of object exploration (i.e. time spent sniffing each object or rearing on it) was scored. Furthermore, to measure the recognition memory, an exploratory preference index was calculated by dividing the amount of time spent exploring the novel object by the total time of objects exploration during the second trial [40, 41].

#### Cued fear-conditioning test

Cued fear-conditioning [42] was used to investigate the ability of test mice to learn and remember an association between conditioned and innocuous stimulus, CS (auditory cue of 2 kHz at 86 dB, in our case) and unconditioned and aversive stimulus, US (electric foot-shock of 0.7 mA). The apparatus consisted of a soundproof cubicle (55 × 60 × 57 cm) hosting a chamber (17 × 17 × 25 cm; Ugo Basile 7532, Comerio, Italy) used for test. The grid-floor of the chamber (steels, 0.2 cm diameter and spaced 0.5 cm) was connected to a shock generator scrambler (Ugo Basile, conditioner 7531). The test consisted of three phases: conditioning, tone and extinction. During the conditioning phase, test mice were individually placed into the test chamber and, following a 180 s acclimation period to the chamber, were subjected to three CS, each of 30 s. During the last 2 s of each CS, mice received a US. Each CS was separated from the following by 95 s of inter-trial interval (ITI) without presentation of either tone or shock. The tone phase was performed after 24 h. Each test mouse was placed into the same chamber and after 180 s of acclimation was subjected to seven CS, each of 30 s. Each CS was separated from the following by an ITI of 10 s. After 24 h, each mouse was subjected to the extinction phase in the same chamber where conditioning and tone phase were performed. Using the same order of the tone phase, mice were subjected to five CS. At the end of each experimental session, the cage was cleaned with 30% ethanol/water solution. Duration of freezing behaviour during the tone phase was measured as an index of emotional memory [43, 44].

#### Hot-plate test

The hot-plate test evaluates thermal pain reflexes due to footpad contact with a heated surface [45]. We performed this test to assess baseline pain reactivity in BALB/cJ mice and control for the possibility that inter-individual differences in the social transmission of pain were associated to a differential pain sensitivity. The apparatus consisted of a metal plate 25 × 25 cm (Socrel Mod. DS-37, Ugo Basile, Italy) over which a transparent Plexiglas cylinder (20 cm diameter; 40 cm height) was placed. During the test, when the metal plate was heated to 55 ± 0.1°C, each mouse was individually placed within the Plexiglas cylinder, onto the metal plate and the latency to the first hind paw-licking or/and front paw-licking or/and jumping behaviours was measured. The test was terminated if the latency exceeded the cut-off time of 60 s [46]. The metal plate and Plexiglas cylinder were cleaned with 30% ethanol/water solution for each new test mouse.

### Experiment 1: Physiological investigations

#### Response to restraint stress

To collect plasma samples for the evaluation of individual hormonal stress reactivity, mice were bled before and after a 25-min restraint stress. Specifically, mice were taken from their home cage, carried by a familiar experimenter to an adjacent room and bled (t0) from the tail (approximately 0.03 ml collected into prechilled ethylenediamine tetraacetic acid (EDTA)-coated tubes; Microvette, Sarstedt, Sevelen, Switzerland) by tail incision [47, 48]. Thus, in total, we collected a maximum of 0.15 ml per mouse corresponding to 0.243 g (considering blood weight equal to 1.062 g/ml). The latter, in turn, corresponds to 1.05% of mouse average body weight. The time elapsed between the experimenter entering the room and the completion of baseline blood sampling was less than 2 min. Subsequently, mice were placed in a transparent Plexiglas restraint tube (2.8 cm diameter) for 25 minutes to induce a stress response. After 25 min, a second sample was taken from the same tail incision (t25). After this sampling, mice were relocated to their home cage. Additional samples were taken 35, 95 and 155 minutes later (t60, t120 and t180) by transporting the mice again to the adjacent room and bleeding them from additional tail incisions. Blood was sampled between 2:30 and 5:30 pm. Samples were cool centrifuged (2500 rpm for 15 min) and the plasma stored at ‒ 80°C until assayed. Corticosterone was measured by a commercial radioimmunoassay (RIA) kit (MP Biomedicals Inc., CA, USA).

#### Trunk blood collection

At the end of all experimental procedures, we collected an additional blood sample to evaluate basal concentrations of testosterone and corticosterone and their ratio. Trunk blood samples were collected by decapitation in heparinized tubes. Blood samples were cool centrifuged at 2500 rpm for 15 minutes to obtain cell free plasma and then frozen at ‒ 80°C until they were assayed for corticosterone and testosterone [49]. Corticosterone and testosterone were measured by a commercial enzyme-linked immunosorbent assay (ELISA) kit (MyBioSource, San Diego, CA, USA).

#### Collection of brain areas

Immediately after trunk blood collection, the brain was rapidly removed and prefrontal cortex, hypothalamus, hippocampus and striatum were collected in Eppendorf tubes and then frozen at ‒ 80°C [50] until they were assayed for BDNF, oxytocin and vasopressin. The evaluation of BDNF peptides was carried out with a commercial ELISA kit (BDNF EmaxTM ImmunoAssay System, by Promega, Madison, WI, USA), following the instructions provided by the manufacturer. Oxytocin (OT) and vasopressin (VP) peptides levels were determined by enzymeimmunoassay (EIA; Oxytocin EIA kit and Arginine Vasopressin EIA kit, respectively, purchased from Cayman Chemical Company, Ann Arbor, MI, USA), following the instructions provided by the manufacturer. Levels of receptors were determined by western blotting analyses for TrkB (BDNF receptor), OT-R (oxytocin receptor), V1a and V3 (vasopressin receptors) using the following antibodies: anti-TrkB (dilution: 1:1000, provided by Cell Signalling, Beverly, MA, USA, catalogue number: 4603), anti-Oxytocin-R (H-60) (dilution 1:1000, provided by Santa Cruz, CA, USA, catalogue number: sc-33209), anti-AVP Receptor V1a (H70) (dilution 1:1000, provided by Santa Cruz, CA, USA, catalogue number: sc-30025), anti AVP Receptor V3 (H90) (dilution 1:1000, provided by Santa Cruz, CA, USA, catalogue number: sc-30026). TrkB, OT-R, V1a and V3 were measured in prefrontal cortex, striatum, hypothalamus and hippocampus of ECP and ECR mice.

### Experiment 2

#### Emotional contagion test: Selection of emotional contagion prone (ECP) and emotional contagion resistant (ECR) mice

Thirty-eight BALB/cJ male mice were used for this experiment. Upon arrival, mice were left undisturbed for 18 days; then, they were all subjected to the emotional contagion test, described in EXPERIMENT 1, at seven weeks of age. As in EXPERIMENT 1, this first test allowed us to identify and select, within the 28 mice that acted as Observers, two subgroups exhibiting extreme values (very high or very low) of emotional contagion, defined as “Emotional Contagion Prone” (ECP group, i.e. upper quartile) and “Emotional Contagion Resistant” (ECR group, i.e. lower quartile).

#### Formalin test

We then assessed, at 15 weeks of age, the pain response of ECP and ECR mice to a classical chemical pain test (formalin test in isolation; see [34, 51] for details). We performed this test to assess baseline chemical pain reactivity in ECP and ECR mice and to control for the possibility that inter-individual differences in the social transmission of pain were explained by differential formalin pain sensitivity [52–54].

Each mouse received a subcutaneous injection in the plantar surface of formalin (25 μl of 1% formalin concentration) and was then placed individually in a glass cylinder (14 cm diameter; 16 cm height). Since subjects had been already injected with formalin (25 μl of 0.2%) in the emotional contagion test, here the contralateral hind paw was used. We scored the duration of paw-licking behaviour during the 30-min test. Mice were tested in the absence of conspecifics in the experimental room. The glass cylinder was cleaned with 30% ethanol/water solution for each new test mouse.

### Experiment 3

Thirty-eight BALB/cJ male mice were used to investigate whether olfactory cues are sufficient to elicit social transmission of pain. Half of these mice were evaluated in a different version of the emotional contagion test: the transparent and perforated Plexiglas partition dividing the Observers (O-OLF, N = 14) from the Demonstrators was replaced by an opaque plastic wall, which allowed olfactory communication but prevented visual contact. By contrast, the remaining half of the Observer mice (O-VIS, N = 14) were assessed in the same version of the test adopted in experiments 1 and 2, namely in both olfactory and visual communication with the Demonstrators. Except for the presence of an opaque instead of transparent partition, testing was performed in identical conditions and following the procedure described in EXPERIMENT 1. Specifically, O-OLF subjects received a subcutaneous injection of 0.2% formalin concentration in the olfactory but not visible presence of a D subject receiving a 1.0% formalin injection. O-VIS subjects received a similar injection (0.2% formalin concentration) in the presence of a D subject receiving a 1.0% formalin injection and providing both olfactory and visual cues. Mice were tested at 14 weeks of age and they were subsequently scored for the duration of paw-licking behaviour during the 30-min test.

### Statistical analysis

To confirm the validity of the emotional contagion test, we first compared the pain response exhibited by Observer (O) and Control (CTRL) mice through repeated measures ANOVA with social context (i.e. visual contact with the Demonstrator mouse vs visual isolation) as between-subjects factor and repeated measurements (six 5-min intervals) as within-subject factor. We then compared pain response exhibited by Observer (O) and Demonstrator (D) mice, with formalin dose (i.e. 0.2% vs 1% concentration respectively) as between-subjects factor and repeated measurements (six 5-min intervals) as within-subject factor. Post hoc analyses were performed through Tukey HSD test.

The experimental design entailed the selection of two subgroups of mice characterized by high and low levels of emotional contagion behaviour. Subgroups were obtained by discretising Observer mice, identified through the time spent in paw-licking behaviour, in quartiles (i.e. the 4 subsets of data values whose boundaries are the 3 quartile points Q_1_, Q_2_, Q_3_). Mice whose values fell within the interquartile range (IQR = Q_3_ –Q_1_) were excluded from further analyses [55–60]. The upper quartile was defined as the “Emotional Contagion Prone” (ECP) group while the lower quartile was defined as the “Emotional Contagion Resistant” (ECR group).

The following analyses were performed using ANOVA with subgroups (2 levels: ECP vs ECR) as factor: time spent in affiliative or aggressive behaviours in the resident-intruder test; time spent in each compartment and number of crossing between compartments in the social approach test; the exploratory preference index in the novel object recognition test; time spent freezing in the cued fear-conditioning; latency to the first paw-licking and/or jumping in the hot-plate test. For analysis of plasma levels of corticosterone in response to restraint stress, the model included 2 subgroups × 5 time points (t0, t25, t60, t120 and t180). In addition, statistics were also performed on the area under the curve, calculated using the trapezoidal rule using ANOVA. The analysis of data from brain and plasma sampling (BDNF, oxytocin, vasopressin and their receptors, corticosterone and testosterone) was performed using ANOVA with subgroup as factor.

All statistical analyses were conducted using the software Statview 5.0 (Abacus Concepts, USA). Data are always expressed as mean ± SEM. Statistical significance threshold was set at p < 0.05. Outlier values, considered as values outside reference interval (mean ‒ 1.96*SD; mean + 1.96*SD), were always excluded. This procedure resulted in the removal of one subject in the resident-intruder test and one subject in the cued fear-conditioning test. No outliers were detected in all other behavioural test paradigms. NS: not significant.

## Results

### Experiment 1: Behavioural tests

#### Social modulation of pain response in the emotional contagion test

As expected, Demonstrator (D) mice, which were tested in response to 1.0% formalin concentration, showed the characteristic biphasic curve of the formalin-induced pain response (**Fig 3B**). Specifically, they showed a first peak of paw-licking during the first 5 minutes (early phase) and a second peak between 10 and 20 minutes after the injection (repeated measures: F_(5,45)_ = 6.099; p < 0.01). Control (CTRL) mice, tested in response to 0.2% formalin concentration, showed a large reduction in pain response compared to D mice (formalin dose: F_(1,13)_ = 23.126; p < 0.01, **Fig 3B, inset**) and a single early peak in paw-licking, which steadily declined to near-zero levels immediately afterwards (repeated measures: F_(5,20)_ = 3.049; p < 0.05). In accordance with predictions, Observer (O) and CTRL mice showed a differential response to the painful stimuli (social context: F_(1,33)_ = 17.697; p < 0.01), with O mice exhibiting a four-fold increase in time spent in paw-licking. Thus, these results indicate that the presence of the demonstrator remarkably influenced pain response in O mice. Notably, O mice were injected the same formalin concentration administered to CTRL mice. Yet, in contrast with CTRL mice that were tested in visual isolation, O mice were in visual contact with a D mouse. As expected, O mice exhibited reduced pain response compared to D mice (F_(1,38)_ = 15.321; p < 0.01, **Fig 3B, inset**). Furthermore, O and D mice exhibited indistinguishable levels of paw-licking during the first five minutes of testing.

#### Selection of emotional contagion prone (ECP) and emotional contagion resistant (ECR) mice

**Fig 4:**
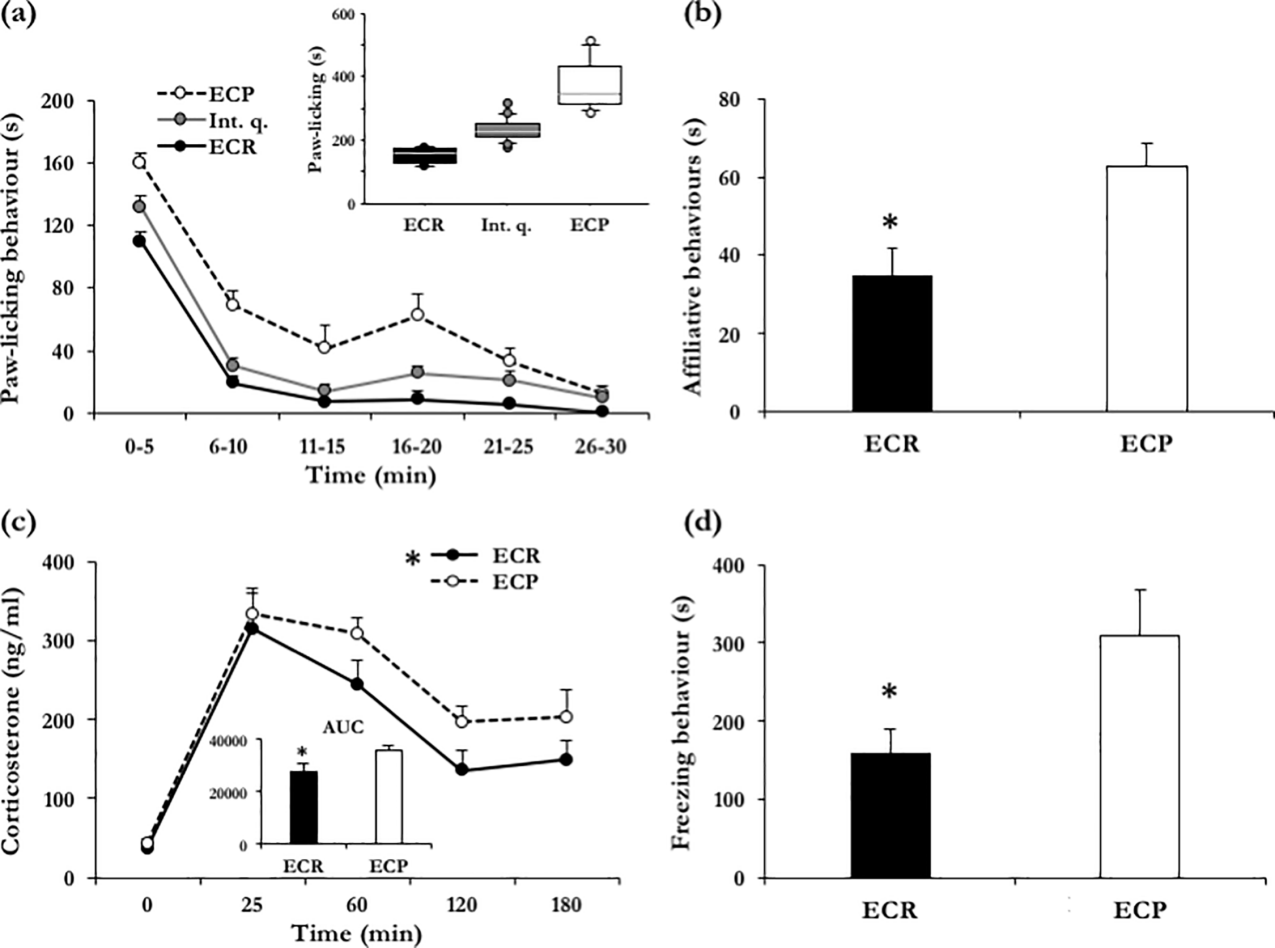
Behavioural results and plasma corticosterone profile following restraint stress. **(a) Identification of subgroups based on time spent in paw-licking behaviour.** Paw-licking behaviour (s) scored in the emotional contagion test by mice belonging to the lower (25%), intermediate (50%) and upper quartiles (25%). The former and the latter constituted, respectively, the emotional contagion resistant (ECR) and the emotional contagion prone (ECP) subgroups. Mice whose values were comprised in the two intermediate quartiles (25% below and 25% above the median) were excluded from further analyses. **(b) Social behaviour.** Affiliative behaviours scored in the resident-intruder test by mice belonging to ECR and ECP subgroups. Data are expressed as seconds out of 10-min testing. * p < 0.05, ECR vs ECP. **(c) Plasma corticosterone profile.** Plasma corticosterone concentrations before a 25-min restraint stress (t0), immediately after (t25) and 35, 95 and 155 minutes later (t60, t120, t180). **(inset)** Comparison for the area under the curve (AUC) response, as a measure of corticosterone release in response to restraint stress, between mice belonging to ECR and ECP subgroups. * p < 0.05, ECR vs ECP. **(d) Emotional memory in the cued fear-conditioning test.** Comparison of time spent in freezing (in the cued fear-conditioning test) between subjects belonging to ECR and ECP subgroups. Data are expressed as seconds out of 10-min testing. * p < 0.05, ECR vs ECP.

To evaluate whether individual differences in emotional contagion correlate with the other phenotypes mapping onto the social behaviour network, Observer mice (N = 30) were assigned to the ECP and ECR subgroups by ranking the set of data values into the 3 quartile points (Q1, Q2, Q3) that divided the data set into 4 equal groups, each group comprising a quarter of the data [55]. Specifically, this ranking was accomplished by taking into account time spent in paw-licking behaviour (s) recorded during the test phase of the emotional contagion test. Animals belonging to the 25% of the extremities, above or below the median, were selected as mice with either ECP or ECR levels, respectively [55]. In other words, the lower and the higher quartiles constituted, respectively, the ECR (N = 7) and the ECP (N = 7) subgroups and were then used for the other phenotypic assessments. Mice whose values fell within the interquartile range (N = 16) were excluded from further analyses (**Fig 4A**).

#### Body weight monitoring

Animals’ body weight was constantly monitored during the whole test battery. Body weight gain was similar in ECR and ECP subgroups (F_(1,12)_ = 3.105, NS; **Table 1**).

**Table 1:** Behavioural and steroid hormonal profiles of ECR and ECP subgroups.

	ECR	ECP	F (df)	p
**Latency to 1**^**st**^ **paw-licking (s)** ^**a**^	10.286 ± 0.714	9.714 ± 0.680	0.336 (1,12)	0.5731
**Body weight (g)**	22.583 ± 0.634	23.695 ± 0.624	3.105 (1,12)	0.1035
**Frequency of crossing** ^**b**^	23.000 ± 4.158	17.000 ± 2.390	1.565 (1,12)	0.2347
**Exploratory preference index** ^**c**^	0.574 ± 0.012	0.656 ± 0.037	4.495 (1,12)	0.0555
**Basal corticosterone (ng/ml)**	37.515 ± 10.952	43.837 ± 10.296	0.177 (1,12)	0.6815
**Basal testosterone (ng/ml)**	1.478 ± 0.434	6.566 ± 3.025	2.771 (1,12)	0.1218
**Testosterone/corticosterone ratio**	0.123 ± 0.060	0.149 ± 0.055	0.102 (1,12)	0.7548

^**a**^ Latency (s) to the first paw-licking in the hot-plate test.

^**b**^ Frequency of crossing during the habituation phase (first 20 minutes of test) of the social approach test.

^**c**^ Exploratory preference index calculated by dividing the amount of time spent exploring the novel object by the total time of object exploration during the second trial in the novel object recognition test. Data are expressed as mean ± SEM.

#### Resident-intruder test

In contrast with our expectations, aggressive behaviour was negligible in this test whereby it accounted for 3.43% of total time. The time-budget of tested subjects was then devoted partly towards non-social activities and partly towards social affiliative behaviours. Considering the durations of anogenital sniffing and received grooming, ECR mice were characterized by a significant reduction in affiliative behaviours compared to ECP subjects (F_(1,11)_ = 9.576, p < 0.05; **Fig 4B**).

#### Social approach test

Social interest was then further investigated in the social approach test. During the habituation phase to the test apparatus, we observed that general locomotion, measured as the frequency of crossing behaviour, did not differ between ECR and ECP subjects (F_(1,12)_ = 1.565, NS; **Table 1**). In the sociability phase, as expected, when experimental mice were allowed to freely choose whether to spend time in the empty compartment or in proximity of a stranger mouse, all mice showed a preference for the latter (F_(1,12)_ = 18.033; p < 0.01). During the preference for social novelty phase, when experimental mice were faced with a choice between two social stimuli differing for the degree of familiarity, subjects spent an undistinguishable amount of time in the two compartments (F_(1,12)_ = 1.903, NS). ECR and ECP subgroups did not differ for social preference either during the sociability (F_(1,12)_ = 1.712, NS) or the social novelty phase (F_(1,12)_ = 0.025, NS).

#### Novel object recognition test

To investigate whether memory impairments were selectively directed towards emotional stimuli or generalized to neutral targets, we performed the novel object recognition test. An exploratory preference index was calculated by dividing the amount of time each mouse spent exploring the novel object by the total time of objects exploration. ECR and ECP subjects did not differ for this preference (F_(1,12)_ = 4.495, NS; **Table 1**).

#### Cued fear-conditioning

ECP and ECR mice were assessed for memory in the cued fear-conditioning test, which is based on emotional modulation of performance by an aversive stimulus. As expected, the conditioning procedure resulted in the formation of fear memory. Thus, during tone presentation, all subjects consistently exhibited freezing behaviour. Notably, ECR subjects showed a significant reduction in time spent in conditioned freezing response compared to the ECP subgroup (F_(1,11)_ = 5.478, p < 0.05; **Fig 4C**).

#### Hot-plate test

To evaluate whether individual differences in emotional contagion behaviour were explained by differential basal pain sensitivity, ECP and ECR mice were tested in the hot-plate paradigm. As reported in **Table 1**, ECR and ECP showed indistinguishable pain sensitivity expressed as the first latency to paw-licking (F_(1,12)_ = 0.336, NS).

### Experiment 1: Physiological investigations

#### HPA function

With the aim of investigating underlying changes in HPA axis function in ECP vs ECR subgroups, mice were tested for physiological response to restraint stress and levels of blood corticosterone measured. As shown in **Fig 4D**, both groups of mice exhibited similar basal corticosterone concentrations and a prompt physiological response to restraint-stress (time interval: F_(4,48)_ = 30.905, p < 0.01). Yet, corticosterone release in response to restrain stress varied between ECR and ECP subjects (subgroup: F_(1,12)_ = 7.983, p < 0.05) with the former exhibiting significantly lower concentrations than the latter.

Finally, analysis of the integral response to restraint stress (area under the curve, calculated using the trapezoidal rule) revealed a similar pattern (F_(1,12)_ = 4.888, p < 0.05; **Fig 4D, inset**). In particular, ECR mice showed decreased area under the curve response compared to ECP subjects.

#### Trunk blood

At the end of testing, we collected trunk blood samples to evaluate testosterone, corticosterone and their ratio. ECR and ECP subjects were indistinguishable in all these parameters (F_(1,12)_ = 2.771, NS; F_(1,12)_ = 0.177, NS; F_(1,12)_ = 0.102, NS, respectively; **Table 1**).

#### BDNF

**Fig 5:**
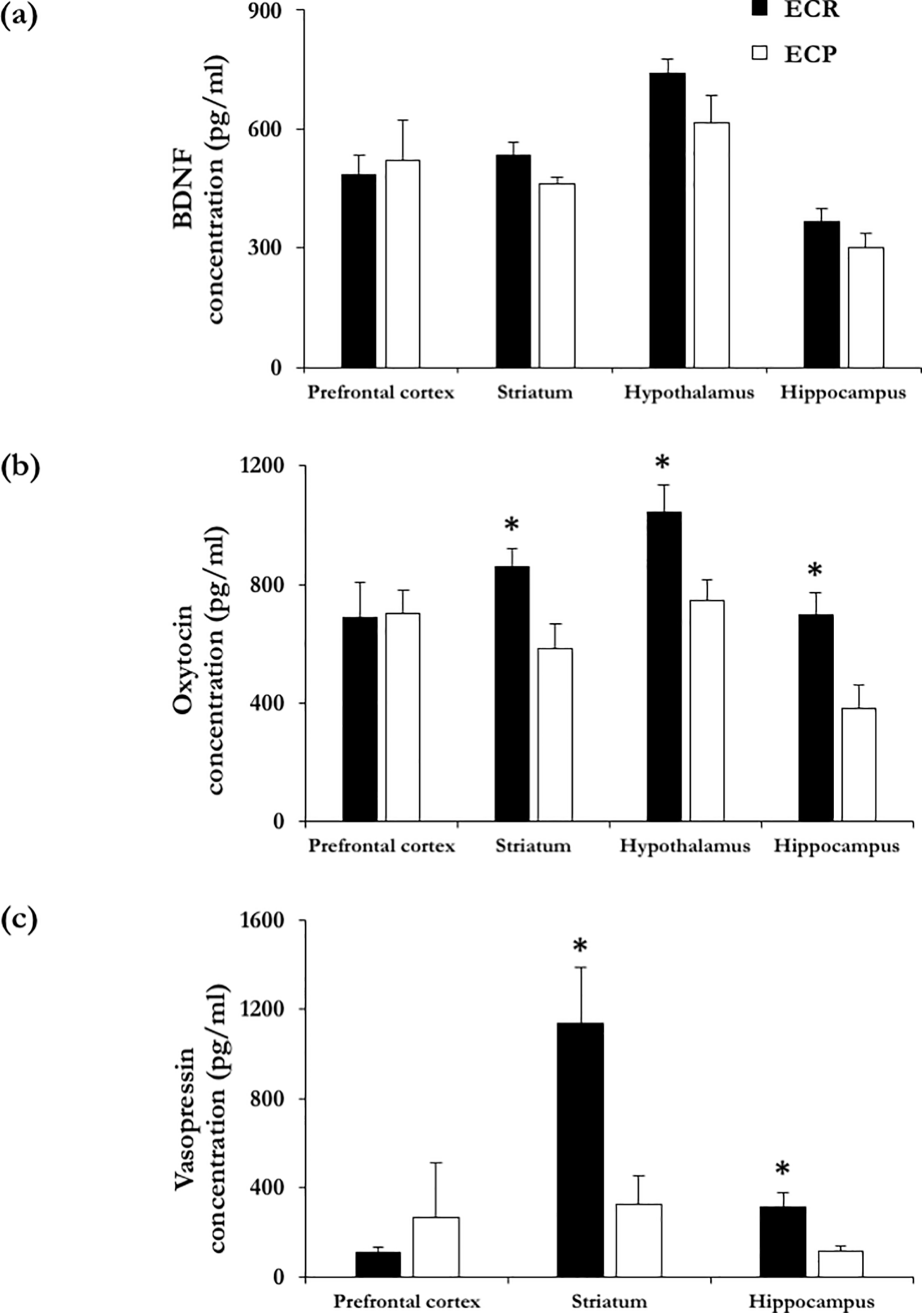
Evaluation of the neuropeptides BDNF, oxytocin and vasopressin. **(a) BDNF concentrations in selected brain areas.** Levels of BDNF measured in prefrontal cortex, striatum, hypothalamus and hippocampus, in mice belonging to emotional contagion resistant (ECR) and emotional contagion prone (ECP) subgroups. **(b) Oxytocin concentrations in selected brain areas.** Levels of the neuropeptide oxytocin measured in prefrontal cortex, striatum, hypothalamus and hippocampus, in mice belonging to ECR and ECP subgroups. * p < 0.05, ECR vs ECP. **(c) Vasopressin concentrations in selected brain areas.** Levels of the neuropeptide vasopressin measured in prefrontal cortex, striatum and hippocampus, in mice belonging to ECR and ECP subgroups. * p < 0.05, ECR vs ECP.

As shown in **Fig 5A**, levels of BDNF in prefrontal cortex, hypothalamus, hippocampus and striatum were indistinguishable in ECR and ECP subgroups (F_(1,12)_ = 0.099, NS; F_(1,12)_ = 2.478, NS; F_(1,12)_ = 1.628, NS; F_(1,12)_ = 3.437, NS, respectively).

**Fig 6:**
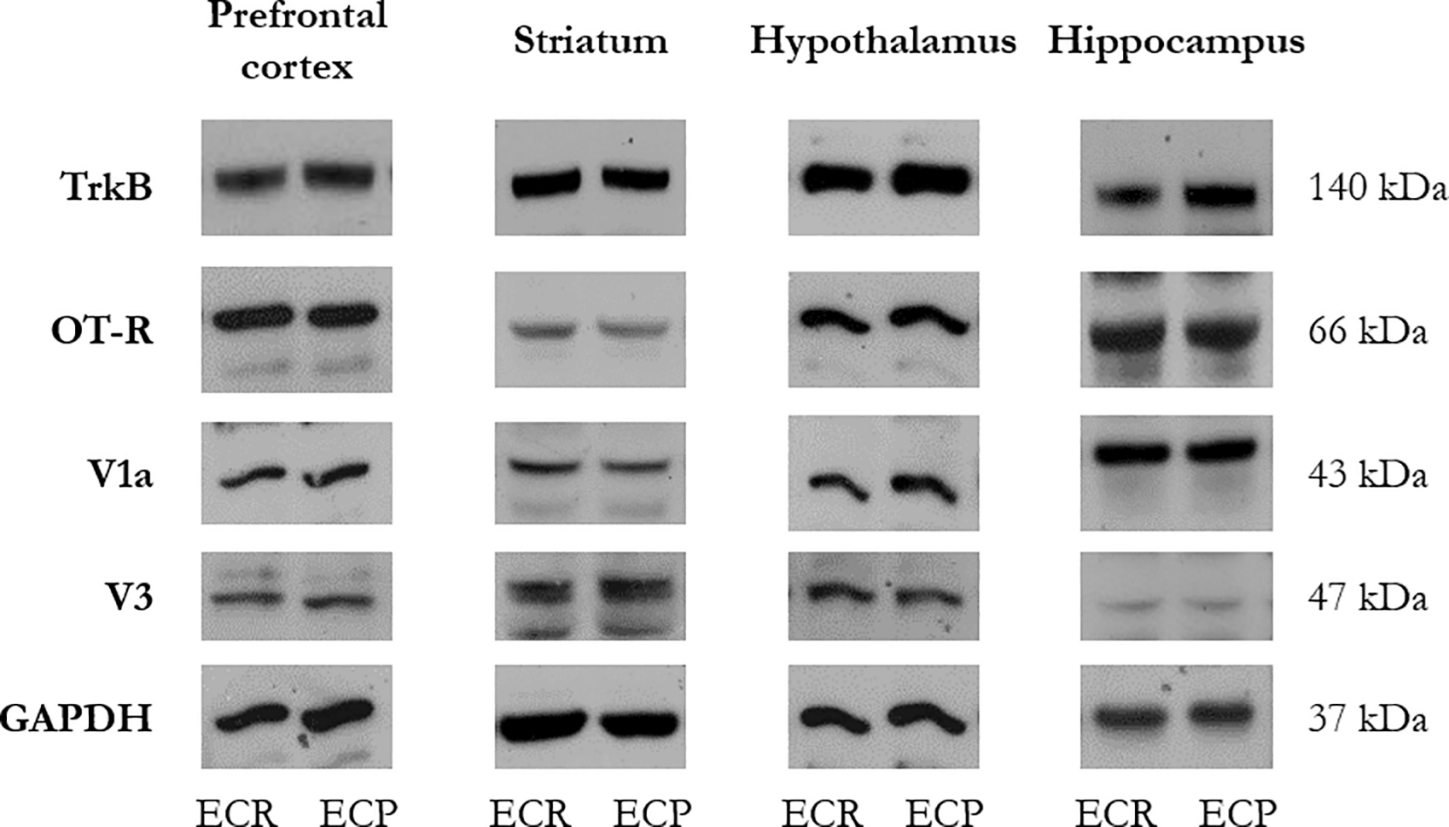
Representative western blots of BDNF, oxytocin and vasopressin receptors. The figure shows representative western blots of TrkB (BDNF receptor), OT-R (oxytocin receptor), V1a, V3 (vasopressin receptors) and GAPDH (for normalization) with the corresponding molecular weight (expressed in kDa), in the prefrontal cortex, striatum, hypothalamus and hippocampus of mice belonging to emotional contagion resistant (ECR) and emotional contagion prone (ECP) subgroups.

Levels of the BDNF receptor (TrkB) were assessed in the same brain areas. As shown in **Table 2**, levels of TrkB in prefrontal cortex and striatum were similar in ECR and ECP subgroups (F_(1,12)_ = 1.147, NS; F_(1,12)_ = 2.640, NS, respectively). For the hypothalamus and hippocampus, levels of TrkB were significantly higher in ECP then ECR mice (F_(1,11)_ = 8.555, p < 0.05; F_(1,11)_ = 5.665, p < 0.05, respectively). Representative western blots of TrkB are shown in **Fig 6**.

**Table 2:** Density of TrkB (BDNF receptors), OT-R (oxytocin receptors), V1a and V3 (vasopressin receptors) in mice belonging to ECR and ECP subgroups.

		ECR	ECP	F (df)	p
**TrkB**	Prefrontal cortex	1.690 ± 0.107	1.949 ± 0.217	1.147 (1,12)	0.3053
	Striatum	0.845 ± 0.80	0.671 ± 0.071	2.640 (1,12)	0.1302
	Hypothalamus	1.148 ± 0.072	1.490 ± 0.094	8.555 (1,11)	0.0138*
	Hippocampus	0.650 ± 0.048	0.861 ± 0.071	5.665 (1,11)	0.0365*
**OT-R**	Prefrontal cortex	0.536 ± 0.025	0.585 ± 0.057	0.608 (1,12)	0.4507
	Striatum	0.467 ± 0.054	0.447 ± 0.053	0.069 (1,12)	0.7976
	Hypothalamus	0.575 ± 0.042	0.550 ± 0.053	0.140 (1,12)	0.7144
	Hippocampus	0.731 ± 0.060	0.779 ± 0.073	0.258 (1,12)	0.6209
**V1a**	Prefrontal cortex	0.881 ± 0.121	0.769 ± 0.220	0.197 (1,12)	0.6648
	Striatum	0.489 ± 0.132	0.352 ± 0.039	0.987 (1,12)	0.3401
	Hypothalamus	0.565 ± 0.045	0.775 ± 0.159	1.599 (1,12)	0.2301
	Hippocampus	0.614 ± 0.056	0.675 ± 0.085	0.359 (1,12)	0.5600
**V3**	Prefrontal cortex	0.910 ± 0.079	0.905 ± 0.276	0.000 (1,12)	0.9839
	Striatum	0.579 ± 0.108	0.603 ± 0.090	0.030 (1,12)	0.8662
	Hypothalamus	0.642 ± 0.053	0.623 ± 0.091	0.033 (1,12)	0.8581
	Hippocampus	0.551 ± 0.071	0.639 ± 0.095	0.555 (1,12)	0.4707

Density of receptors (normalized against Glyceraldehyde-3-phosphate dehydrogenase, GAPDH) in relevant brain areas (prefrontal cortex, striatum, hypothalamus and hippocampus) in ECR and ECP mice. Data are expressed as mean ± SEM.

* p < 0.05 ECR vs ECP.

#### Oxytocin and vasopressin

As shown in **Fig 5B**, in the absence of significant variations in the prefrontal cortex (F_(1,12)_ = 0.009, NS), ECR mice showed increased concentrations of oxytocin in striatum, hypothalamus and hippocampus (F_(1,11)_ = 7.192, p < 0.05; F_(1,12)_ = 6.689, p < 0.05; F_(1,11)_ = 8.511, p < 0.05, respectively). Furthermore, compared to ECP, ECR mice showed increased concentrations of vasopressin in striatum and hippocampus (F_(1,10)_ = 9.741, p < 0.05; F_(1,11)_ = 7.572, p < 0.05, respectively; **Fig 5C**). Yet, vasopressin concentrations in ECP and ECR mice did not differ in prefrontal cortex (F_(1,7)_ = 0.983, NS). Finally, irrespective of changes in peptide concentrations, receptor levels (OT-R, V1a and V3) in the same brain areas (**Table 2**), did not differ between ECP and ECR mice. Representative western blots of OT-R, V1a and V3 are shown in **Fig 6**.

### Experiment 2

#### Emotional contagion test: Selection of emotional contagion prone (ECP) and emotional contagion resistant (ECR) mice

Observer mice (N = 28) were assigned to the ECR and ECP subgroups by ranking time spent in paw-licking behaviour (s) recorded during the test phase of the emotional contagion test. Mice belonging to the lower and the higher quartiles constituted, respectively, the ECR (N = 7) and the ECP (N = 7) subgroups whilst the remaining mice (N = 14) were excluded from the subsequent phenotypic assessments [55]. For further details, please see the corresponding paragraph in the section “EXPERIMENT 1”.

#### Formalin test

To evaluate whether subjects differing along emotional contagion behaviour differ in terms of baseline pain response to a chemical agent, ECR and ECP mice were tested in the classical formalin test. As expected, they showed indistinguishable pain sensitivity expressed as duration of paw-licking behaviour (F_(1,12)_ = 2.88, NS; ECR: 37.00 ± 7.91 s and ECP: 59.29 ± 10.48 s; N = 7 per group).

### Experiment 3

Mice tested with the transparent Plexiglas partition (O-VIS: 54.95 ± 8.43 s; N = 14) exhibited indistinguishable levels of paw-licking behaviour than those tested with the opaque plastic wall (O-OLF: 59.16 ± 7.95; N = 14; F_(1,26)_ = 0.13; NS).

Thus, these results indicate that the mere presence of olfactory cues provided by the Demonstrator, with or without visual contact, remarkably influenced the social transmission of a painful experience between individuals. Notably, O-VIS mice were injected the same 0.2% formalin concentration administered to O-OLF mice. Yet, in contrast with O-OLF mice that were tested in olfactory contact but in visual isolation (because of the opaque partition), O-VIS mice were also in visual contact with a D mouse.

## Discussion

The main aim of the present study was to demonstrate the association between emotional contagion and other behavioural components of the social behaviour network [22]. To this aim, we performed an individual-differences study in BALB/cJ mice. Compared to other strains, BALB/cJ mice are characterized by a significant reduction in sociability and observational fear learning and by a dampened physiological reactivity to stressors [7, 61–63]. Since we aimed at modelling a pathological lack of empathy (callousness), this specific inbred strain allowed us to obtain, in ECR mice, extremely low levels of a rudimentary form of empathy-like behaviour. We also selected an inbred strain with the aim of limiting the number of subjects used. Indeed, inbred strains are characterised by a reduced inter-individual phenotypic variation [64] which, in turn, translates into an increased statistical power of experimental data [65].

The interest in the association between empathy and the other aforementioned factors is manifold: first, empathy is a crucial cognitive-emotional domain directly involved in survival and reproductive success [14]; second, it may bestow heuristic advancements in terms of the biological determinants of social behaviour [8]; finally, lack of empathy constitutes a crucial symptom in several personality disturbances, including CD [66].

To achieve our aims we assessed social and aggressive behaviour, emotional memory, physiological stress reactivity and neurobiological regulations in selected brain areas, in laboratory mice selected for extremely high or low levels of emotional contagion capabilities. We believe that a model of CD shall incorporate the concept of behavioural syndrome, defined as “a suite of correlated behaviours expressed either within a given behavioural context […] or across different contexts” [67], whereby it bridges evolutionary adaptive considerations with applied experimental psychobiology. We propose that, in the context of CD, an experimental model could be considered as a behavioural syndrome in which a given personality is characterised by co-occurring excess values among a set of given variables, mapping onto clinical CD: aggression, callousness and shallow or deficient affect [68].

We observed that trait differences in emotional contagion extend to several behavioural and physiological domains and are mediated by specific neurobiological adaptations within the “social behaviour network” [22]. Collectively, our data show that reduced exhibition of pain in the presence of a suffering familiar conspecific is associated with a behavioural syndrome of reduced sociability, impaired memory for negative events, reduced physiological reactivity to stressors and increased concentrations of central oxytocin and vasopressin. In contrast, basal pain sensitivity, body weight gain, locomotor behaviour, non-emotional related recognition memory and testosterone/corticosterone ratio were equivalent in both subgroups of mice. We propose that our findings may model those CU traits that constitute a crucial symptom in several personality disturbances, including CD [66].

### Inter-individual differences in emotional contagion are independent of basal pain sensitivity

The identification of emotional contagion rested upon an established experimental paradigm [19] evaluating the extent to which the perception of pain in response to a nociceptive stimulus can be socially modulated. To this aim, we compared the exhibition of pain in isolated individuals, injected with formalin, with that exhibited by experimental subjects, treated with the same dose of formalin, in the presence of a demonstrator experiencing a higher level of pain [69]. Based on available literature indicating that the degree of familiarity represents a crucial factor capable of mediating the social transmission of pain [10, 19, 20, 70], demonstrators and observers were related (i.e. cagemates). Our findings in mice are consistent with the perception-action model of empathy-like behaviour proposed by Preston and de Waal [8]. According to this model, the perception of a given state in another individual activates the subject’s corresponding representations, thereby culminating in the expression of somatic and autonomic responses (paw-licking behaviour). Following the conduction of the emotional contagion test in a cohort of mice, we identified two subgroups (the lower and the upper quartile) exhibiting very low or high emotional contagion. The study of subgroups identified through this approach has represented a useful tool in several investigations aimed at identifying the role of a given variable on the exhibition of other phenotypes in humans and experimental models (for humans, [56, 57, 60]; for rodents, [58, 59]).

Since the experimental paradigm adopted to identify the two subgroups involves the exhibition of pain in response to a noxious stimulus, we addressed whether basal differences in emotional contagion behaviour were dependent on differential basal pain sensitivity. To this aim, we investigated whether ECR and ECP mice differed for other indicators of pain reactivity [45]. Since it has been shown that pain tests of different modalities (e.g. thermal vs chemical) do not correlate well [52–54], we performed both a hot-plate test and a formalin test to demonstrate equal pain sensitivities in ECP and ECR mice. We observed that ECR and ECP mice exhibited an indistinguishable pain response to both a thermal and a chemical agent, thereby strengthening the view that inter-individual differences in emotional contagion behaviour were unlikely to be mediated by differential basal pain sensitivity.

### Low emotional contagion is associated with impaired emotional memory and reduced sociability

Following the selection of the experimental subgroups, we addressed whether individual variations in emotional contagion related to a differential expression of social and aggressive behaviour and emotional processing. The focus on these phenotypes depends on the fact that they map on overlapping brain structures [22] and that their simultaneous aberrant expression contributes to the nosography of CD [24, 66]. One of the key aspects of CD is a general increase in aggressive behaviour associated with very limited prosocial emotions [25]. We thus anticipated ECR individuals to display increased aggression compared to ECP subjects. To test this hypothesis, we performed a resident-intruder test adopting a protocol that has been shown to elicit bursts of aggressive behaviour in different strains of mice (e.g. [36]). However, in contrast with available literature, we failed to observe consistent aggressive behaviours directed from the focal subject to the intruder C57BL/6J mouse and we do not have a conclusive explanation for this very limited expression of aggressive behaviour. While the experimental paradigm did not elicit remarkable aggression, it allowed us to evaluate levels and characteristics of the social interaction. Indeed, in this paradigm, mice exhibited a considerable amount of social behaviours. In line with our expectations, mice of ECR subgroup exhibited very low levels of affiliative behaviours compared to the ECP subgroup. This result confirms a significant association between reduced appraisal of the emotional state of a conspecific and low levels of social affiliation in our mouse model.

In CD, individuals that qualify for the “with limited prosocial emotions” specifier generally show a considerable reduction in sensitivity to punishment and emotionality [66, 71]. To evaluate this aspect, we conducted a cued fear-conditioning test in which mice are requested to form an association between an aversive and an unconditioned stimulus. Specifically, we evaluated the amount of freezing behaviour displayed in response to the unconditioned stimulus (the tone) one day following the conditioning phase. This response has traditionally been considered an index of emotional memory [43, 44]. Compared to ECP, ECR mice showed a significant deficit, thereby strengthening the view that low empathy may relate to a reduced emotionality (namely, capacity for punishment-induced conditioning). This possibility is further substantiated by the observation that ECR and ECP mice showed an indistinguishable performance in the Novel object recognition test, which is based on recognition memory and has a negligible emotional component.

### Reduced emotionality in ECR mice relates to reductions in HPA reactivity to stressors

Clinical evidence suggests that reductions in social behaviour may relate to blunted physiological stress reactivity [72]. Furthermore, data on patients with CD indicate that they may often exhibit reduced cortisol response to psychological stressors [73–75]. To evaluate whether the reduced social behaviour and impaired emotional memory in ECR mice related to altered physiological stress reactivity, we evaluated plasma corticosterone concentrations before and after a 25-min restraint stress. In line with our predictions, ECR mice showed a consistent reduction in corticosterone stress reactivity compared to ECP mice. The association between corticosterone concentrations and emotional memory has been reported in numerous studies [76–78]. For example, Hagewoud and collaborators (2011) observed that an experimental procedure capable of reducing corticosterone response to a stressful stimulus also resulted in impaired memory in fear-conditioning [77]. Likewise, reductions in HPA reactivity to stressors have been reported to relate to impairments in social behaviour [79, 80]. These findings support the association in the mouse model of a unitary behavioural profile, consisting of reduced social affiliation and empathy-like behaviour, as well as a generally blunted functional activation of the physiological stress-response system. These different aspects appear in line with what reported in subjects diagnosed with paediatric CD [25].

### Behavioural and neuroendocrine alterations in ECR individuals relate to alterations in brain neurochemistry

Several authors identified the brain structures underlying the exhibition of empathy-like behaviour [15]. For example, the perception of distress in others has been reported to activate highly conserved brain areas as prefrontal cortex, insula, amygdala, striatum, thalamus and hippocampus [81–84]. Moreover, these brain areas communicate through specific neuropeptides considered “facilitators” of empathic capacity, as oxytocin (OT), vasopressin (VP, [85]), cannabinoids [86, 87], opioids [88–90] and the neurotrophin BDNF [91, 92]. Furthermore, the same brain structures and neurochemical mediators have been involved in the exhibition of the other parameters considered in the present study [93, 94]. Based on these considerations, we investigated the concentrations of OT and VP and their receptors, in brain areas–mediating the phenotypes investigated herein–in both ECR and ECP subjects.

While OT and VP concentrations in prefrontal cortex did not differ between groups, ECR mice exhibited higher concentrations of both neuropeptides in striatum, hypothalamus and hippocampus. Since OT and VP are reported as “facilitators” of empathic behaviour (e.g. [85]) we anticipated observing a reduction rather than an increase in ECR mice. Thus, these results are in disagreement with our original hypothesis. While we cannot fully explain this conflicting finding, we note that available literature in human subjects regarding the role of OT and VP on empathic behaviour as yet failed to provide conclusive evidence. Thus, while in several cases OT has been shown to favour empathy [95–99], in some other situations OT administration has been shown to reduce empathic responses. Abu-Akel and collaborators [100] reported that OT administration increased empathy to pain in humans while evaluating a painful situation adopting the perspective of another individual but not the self. An independent study from the same group [101] revealed that the effects of OT on empathy may depend on whether the subject considers the individuals in pain as belonging to the same group (in-group) or to a different one (out-group). In contrast with the possibility that OT invariably increases empathic responses, Bos and colleagues [96] observed that OT may reduce emotional empathy when addressed in terms of individual response to pain of others. Specifically, through a functional neuroimaging study, the authors investigated the role of intranasal OT in mediating individual responses to the pain of others. The authors first reported a robust activation of insula and sensorimotor regions when volunteers were viewing other subjects in pain and then evaluated the effects of intranasal OT administration on brain activation patters. In contrast with the expectations, brain activation was strongly reduced following intranasal administration of OT [96]. These results are in accordance with our data whereby we also observed that increased concentrations of oxytocin related to a reduced emotional empathy in ECR subjects. There is evidence that the neural circuitries governing pain sensation and emotional empathy for pain are shared [102, 103]. Accordingly, the study conducted by Bos and collaborators (2015) also indicated that the OT-mediated reduction in neural activity involved the pain circuitry; thus, reduced exhibition of empathic responses may be secondary to a reduction in pain perception. The aforementioned studies strengthen the view that the modulatory role of OT on empathy is dependent on the context and on the specific type of empathy under analysis. The multimodal nature of empathic responses has been cogently summarised by De Dreu [104]. In this review manuscript, the author observes that while OT generally increases empathic responses, it may have opposite effects if the suffering conspecific belongs to a different social group. In this case, reduced empathy is apparently due to an OT-mediated increase in in-group cohesion.

Ultimately, the association between the high brain levels of OT and VP and the dampened emotional reactivity exhibited by the ECR subgroup may be related to the fact that OT mediates several phenotypes mapping onto our emotional contagion test. These results shed new light on the effects of OT on empathy by suggesting that this neuropeptide may yield opposite effects depending on the specific type of empathy under consideration. Notably, cognitive empathy and empathy for pain have been known to dissociate in clinical groups [105], with individuals diagnosed for CD, presenting low emotional empathy, but high cognitive empathy.

The neurotrophin BDNF also plays a key role in psychiatric conditions characterized by dysfunction of social behaviour [106–108]. In preclinical models, BDNF hippocampal concentrations have been directly related to changes in emotional behaviour [92]. In our study, levels of BDNF in prefrontal cortex, hypothalamus and hippocampus were similar in ECR and ECP subgroups. Instead, a slight elevation in BDNF was evidenced in the striatum of mice characterized by low empathy. Notably, a corresponding down-regulation in density of TrkB receptors in the ECR hypothalamus and hippocampus confirmed a consistent differential regulation in mice of ECR and ECP subgroups.

### Final remarks

The fact that emotional contagion co-occurred with other phenotypes strengthens the robustness of our data. Specifically, since our experimental population is composed of genetically identical individuals, had emotional contagion been independent of the other phenotypes, the likelihood to observe differences in the latter would have been extremely low. Yet, our data show that extreme values in these behaviours co-occur even in this highly selected population. Therefore, future studies elucidating the factors underlying differences in empathy may point toward the identification of fundamental mechanisms involved in complex behavioural syndromes.

Age, genotypic, or vendor differences cannot explain the type of phenotypic variance identified in the emotional contagion paradigm. Indeed, only 7-week-old male Balb/cJ mice from a single vendor were used. Examples of phenotypic variability in genetically identical (inbred) mice have classically been ascribed to environmental influences that are difficult to control and measure, such as variations in prenatal and postnatal environment (e.g. intrauterine position [109, 110]; early interactions with mother and peers [111]). Experiments performed in inbred mice raised in rigorously defined environments evidenced that a portion of variability in biological variables is related neither to genetic nor to environmental influences [112]. This “third component” to natural variation is hypothesized to maintain Gaussian distributions of biological traits irrespective of environmental influences and sequence constraints and is currently attributed to epigenetic mechanisms, such as histone acetylation or methylation, which regulate gene expression without changing DNA sequence [113, 114]. While these modifications play an important role in stress responses [115, 116], future work is needed to delineate the relative contribution of epigenetic, genetic and environmental factors that may together explain variations in empathy.

## Conclusion

The present study showed that extreme-oriented profiles in emotional contagion, a construct amenable to being addressed in laboratory rodents, are associated with consistent reductions in social behaviour, emotional memory and physiological stress reactivity. Furthermore, these impairments are associated with neurochemical changes in brain pathways underlying the aforementioned phenotypes. Besides contributing fundamental knowledge regarding the biological determinants of the social behaviour network, the present study may thus offer preclinical insights with respect to psychiatric disturbances characterised by lack of empathy. The cohesive phenotypic pattern identified in ECR mice, together with the study of its neurochemical counterparts, may inform future research on the biological determinants of CD and on potential innovative therapeutic approaches.

## Supporting information

S1 File Data sets. The Excel file contains the data sets of the three experiments, organized in three different sheets.
(XLS)
